# Activities of Rifampin, Rifapentine and Clarithromycin Alone and in Combination against *Mycobacterium ulcerans* Disease in Mice

**DOI:** 10.1371/journal.pntd.0000933

**Published:** 2011-01-04

**Authors:** Deepak Almeida, Paul J. Converse, Zahoor Ahmad, Kelly E. Dooley, Eric L. Nuermberger, Jacques H. Grosset

**Affiliations:** 1 Center for Tuberculosis Research, Division of Infectious Diseases, Johns Hopkins University School of Medicine, Baltimore, Maryland, United States of America; 2 Division of Clinical Pharmacology, Johns Hopkins University School of Medicine, Baltimore, Maryland, United States of America; 3 Department of International Health, Johns Hopkins Bloomberg School of Public Health, Baltimore, Maryland, United States of America; The George Washington University Medical Center, United States of America

## Abstract

**Background:**

Treatment of *Mycobacterium ulcerans* disease, or Buruli ulcer (BU), has shifted from surgery to treatment with streptomycin(STR)+rifampin(RIF) since 2004 based on studies in a mouse model and clinical trials. We tested two entirely oral regimens for BU treatment, rifampin(RIF)+clarithromycin(CLR) and rifapentine(RPT)+clarithromycin(CLR) in the mouse model.

**Methodology/Principal Findings:**

BALB/c mice were infected in the right hind footpad with *M. ulcerans* strain 1059 and treated daily (5 days/week) for 4 weeks, beginning 11 days after infection. Treatment groups included an untreated control, STR+RIF as a positive control, and test regimens of RIF, RPT, STR and CLR given alone and the RIF+CLR and RPT+CLR combinations. The relative efficacy of the drug treatments was compared on the basis of footpad CFU counts and median time to footpad swelling. Except for CLR, which was bacteriostatic, treatment with all other drugs reduced CFU counts by approximately 2 or 3 log_10_. Median time to footpad swelling after infection was 5.5, 16, 17, 23.5 and 36.5 weeks in mice receiving no treatment, CLR alone, RIF+CLR, RIF alone, and STR alone, respectively. At the end of follow-up, 39 weeks after infection, only 48%, 26.4% and 16.3% of mice treated with RPT+CLR, RPT alone and STR+RIF had developed swollen footpads. An *in vitro* checkerboard assay showed the interaction of CLR and RIF to be indifferent. However, in mice, co-administration with CLR resulted in a roughly 25% decrease in the maximal serum concentration (Cmax) and area under the serum concentration-time curve (AUC) of each rifamycin. Delaying the administration of CLR by one hour restored Cmax and AUC values of RIF to levels obtained with RIF alone.

**Conclusions/Significance:**

These results suggest that an entirely oral daily regimen of RPT+CLR may be at least as effective as the currently recommended combination of injected STR+oral RIF.

## Author Summary

Buruli ulcer (BU) is found throughout the world but is particularly prevalent in West Africa. Until 2004, treatment for this disfiguring disease was surgical excision followed by skin grafting, procedures often requiring months of hospitalization. More recently, an 8-week regimen of oral rifampin and streptomycin administered by injection has become the standard of care recommended by the World Health Organization. However, daily injections require sterile needles and syringes to prevent spread of blood borne pathogens and streptomycin has potentially serious side effects, most notably hearing loss. We tested an entirely oral regimen, substituting the long acting rifapentine for rifampin and clarithromycin for streptomycin. We also evaluated each drug separately. We found that rifapentine alone is as good as rifampin plus streptomycin, but the simultaneous addition of effective clarithromycin doses, at least in the mouse, reduces the activity of both rifampin and rifapentine, making it difficult to assess the efficacy of the oral regimens in the model. Studies of serum drug concentrations indicated that separating treatment times by one hour or reducing the clarithromycin dose to one active in humans should overcome this issue in experimental and clinical BU treatment, respectively.

## Introduction

*Mycobacterium ulcerans* disease, also known as Buruli ulcer (BU), is the third most prevalent disease caused by mycobacteria [1]. It is characterized by deep and necrotizing skin ulcers with undermined edges resulting from the secretion by *M. ulcerans* of an immunosuppressive macrolide toxin, termed mycolactone [2]. It is predominantly found in scattered foci in tropical riverine and marshy regions throughout the world. In certain parts of Africa its prevalence may exceed 150/100,000 individuals [3].

Until 2004, the recommended treatment for BU was surgical excision and skin grafting [1]. However, experimental studies using the mouse footpad model demonstrated that the combination of rifampin (RIF) and an aminoglycoside was bactericidal for *M. ulcerans* [4], [5], [6], [7]. Based on these findings and subsequent studies in humans [8], [9] the daily administration of the streptomycin-rifampin (STR+RIF) combination for 2 months was recommended by the World Health Organization (WHO) for the treatment of BU [10]. Depending on the size, severity and location of the ulcer, additional surgical intervention with skin grafting was also recommended. Treatment with STR requires intramuscular injection, which is difficult and expensive to implement in resource-poor countries since it requires use of sterile needles and syringes to avoid infection with blood borne pathogens. Therefore, the development of an entirely oral regimen is desirable [11].

*In vitro*, *M. ulcerans* is susceptible to a limited number of oral antibiotics including fluoroquinolones and macrolides [12], [13],[14],[15],[16]. However, in the mouse model, the combination of clarithromycin (CLR), a bacteriostatic or weakly bactericidal drug against *M. ulcerans*, and RIF, which also has limited bactericidal activity [4], [6] has not consistently shown efficacy similar to the standard STR+RIF regimen. Three mouse studies assessing the bactericidal activity and the relapse rate after treatment completion yielded conflicting results. In the first study [5], the oral combination was less effective than the standard aminoglycoside plus RIF combination whereas in the second and third [12], [13] studies, both combinations appeared to be as effective as the STR+RIF controls. There is, therefore, a need to directly address the issue of oral antibiotic treatment of BU, both with RIF and CLR and with new treatment regimens including anti-BU drugs that may have improved activity. Rifapentine (RPT), a rifamycin derivative with a much longer half-life than RIF could be an ideal substitute. In the murine model of tuberculosis when substituted for RIF at 10 mg/kg in a daily regimen in combination with isoniazid and pyrazinamide, it shortened the duration of treatment necessary to achieve cure [17], [18]. Such regimens are currently under evaluation in at least 3 Phase II trials for tuberculosis treatment. In a murine model of *M. ulcerans* disease, daily RPT at the lower dose of 5 mg/kg has also been shown to be as active as, or even more active than, daily RIF at 10 mg/kg [13].

In this study, we hypothesized that the use of daily RPT along with CLR would increase the efficacy of the rifamycin-CLR combination and help in the development of an entirely oral regimen for treatment of BU. We first demonstrated that there were no negative *in vitro* interactions of CLR and RIF (as a representative rifamycin) and then compared the efficacy of the RIF+CLR regimen to that of the RPT+CLR regimen using the STR+RIF standard regimen as control to determine whether daily RPT is a better substitute for daily RIF in the treatment of *M.* ulcerans disease in this murine model and whether daily RPT+CLR is also a better substitute for the standard daily STR+RIF combination.

## Materials and Methods

### Antimicrobials

STR and RIF were purchased from Sigma (St. Louis, MO) and RPT was a gift from sanofi-aventis pharmaceuticals (Paris, France). CLR was a gift from Abbott Laboratories (Abbott Park, U.S.A.). Stock solutions of RIF, RPT and CLR were prepared in sterile 0.05% agarose solution and STR was prepared in sterile normal saline. All stock solutions were prepared weekly and were stored at 4°C. All antimicrobials were administered orally (by gavage) using an esophageal cannula, except STR which was given by subcutaneous injections.

### Bacterial strain

A recent isolate of *M. ulcerans* from a Ghanaian patient, strain *Mu*1059 [19] provided by Dr. Pamela Small, was used for the study.

### *In vitro* checkerboard study

To determine whether the RIF and CLR interaction is synergistic, indifferent or antagonistic, serial two-fold concentrations ranging from 0.125 to 2 µg/ml of both drugs alone and in combination were prepared in 7H11 agar+Oleic Acid-Albumin-Dextrose-Catalase (OADC) supplement. Eight-week-old colonies of *Mu*1059 from 7H11 agar plates were suspended in phosphate-buffered saline (PBS), briefly vortexed, and kept undisturbed for 30 minutes to allow larger particles to settle. The optical density at 600 nm of this suspension was adjusted to 1, and 500 µl of the appropriate dilutions were plated in duplicate on antibiotic-containing plates and control plates without antibiotic. Plates were incubated at 32°C, and final CFU counts were performed after 12 weeks. The MIC was defined as the lowest drug concentration to inhibit growth of at least 99% of CFU on drug-free control plates. The fractional inhibitory concentration (FIC) value of individual drugs was then calculated using the MIC of the drug alone and MIC of the drug in combination. The sums of the two FIC values were combined to give the ΣFIC value which was then used to determine whether synergism (ΣFIC≤0.5), indifference ΣFIC (>0.5 to ≤4) or antagonism (ΣFIC>4) occurred between the antibacterial agents. All calculations were performed in accordance with current accepted standards [20], [21], [22].

### Inoculum preparation for the *in vivo* study

For each infection, an aliquot of a twice-mouse-passaged *Mu*1059 strain stored at −80°C was thawed and inoculated in mouse footpads. Once the footpads were swollen to a lesion index of 2–3 (defined as inflammatory footpad/hind foot swelling) [5], mice were sacrificed and footpad tissue was harvested, minced and suspended in sterile PBS. The solution was vortexed briefly, allowed to stand for 30 minutes, and the supernatant was used for footpad infection. Prior to infection, the inoculum was checked qualitatively for acid-fast bacilli, serially diluted, and plated for CFU counts on Middlebrook selective 7H11 plates (Becton-Dickinson, Sparks, MD).

### Mouse model, infection and treatment

The kinetic method developed by Shepard for assessing the activity of anti-leprosy drugs [4], [5], [6], [23], was used to assess drug activity. In brief, 320 female BALB/c mice aged 4-to-6 weeks (Charles River, Wilmington, MA) were infected in the right hind footpad with 0.03 ml of the *M. ulcerans* suspension. After infection, mice were randomized to one of two control groups or one of six test groups. The control groups included untreated negative controls (n = 50), and mice treated with STR+RIF as positive controls (n = 55). The test groups included mice treated with each antibiotic alone, i.e., CLR (n = 30), STR (n = 25), RIF (n = 25), and RPT (n = 25), and the two-drug combinations RIF+CLR (n = 55) and RPT+CLR (n = 55). Ten mice from the untreated group were sacrificed the day after infection (D1) and 11 days later at treatment initiation (D11) to establish baseline CFU counts in the footpads. All mice were treated for 4 weeks, 5 days per week. The drugs were given at the following doses that are equivalent (similar AUC) to the human doses [25], [26]: RIF 10 mg/kg, RPT 10 mg/kg, STR 150 mg/kg and CLR 100 mg/kg. On treatment completion, 5 mice from each group were sacrificed for quantitative CFU counts in the footpads and all of the remaining mice were kept without treatment to determine the time to footpad swelling.

For quantitative footpad CFU counts, each footpad was harvested after having been thoroughly disinfected with soap and sterile PBS followed by 70% alcohol swabs. The footpad tissue was homogenized by fine mincing and suspended in 2 ml sterile PBS. Appropriate dilutions were plated on selective 7H11 plates and incubated at 32°C for 12 weeks before CFU were enumerated. This study was carried out in strict accordance with the recommendations in the Guide for the Care and Use of Laboratory Animals of the National Institutes of Health. All animal procedures were approved by the Johns Hopkins Animal Care and Use Committee (protocol MO08M240) and conducted according to relevant national and international guidelines.

### Assessment of treatment efficacy

The activity of each treatment was assessed in terms of CFU counts on treatment completion and median time to footpad swelling in treated mice compared with untreated control mice. CFU counts were performed by harvesting and homogenizing the footpad as described above and suspending each footpad in 2 ml PBS. Serial 10-fold dilutions were prepared and 0.5 ml of appropriate dilutions were plated in duplicate on 7H11 selective plates. The plates were then incubated at 32°C for 12 weeks before the CFU counts were made. Median time to footpad swelling was assessed by checking the footpads of mice every week for 39 weeks after infection. If the median time to footpad swelling in treated mice exceeded that in untreated mice by no more than the duration of the treatment, i.e. 4 weeks, then the treatment was considered to be bacteriostatic. Longer median time to swelling was indicative of bactericidal activity or prolonged post-antibiotic effect. Absence of swelling at the end of the follow-up period was indicative of sterilizing potential.

### Pharmacokinetic studies of rifamycin-CLR combinations

Because we observed a negative antimicrobial interaction between both rifamycin derivatives and CLR *in vivo*, a series of single-dose pharmacokinetic (PK) studies were performed in BALB/c mice. In the first study mice were co-administered 100 mg/kg of CLR and RIF 10 mg/kg. One sample for PK analysis was collected per mouse at 1, 2, 4, 6, 9 or 16 hrs after dosing. From these data, composite concentration-time curves were developed and compared. Because RIF serum concentrations appeared to be diminished when the two drugs were dosed together, we conducted a second study in which mice received 10 mg/kg of RIF alone, 10 mg/kg of RIF followed by 100 mg/kg of CLR one hour later, or 10 mg/kg of RIF co-administered with a lower dose of CLR (10 mg/kg). In a third study we substituted RPT for RIF and assessed RPT serum concentrations in mice receiving 10 mg/kg of RPT alone, 10 mg/kg of RPT co-administered with 100 mg/kg of CLR, or 10 mg/kg of RPT followed 1 hour later by 100 mg/kg of CLR. In a fourth study we evaluated RPT serum concentrations after RPT 10 mg/kg was co-administered with CLR at 10 mg/kg. Serum samples were frozen at −80°C and shipped overnight on dry ice to the Infectious Disease Pharmacokinetics Laboratory, National Jewish Medical and Research Center, Denver, CO. Drug concentrations were determined using validated HPLC methods. PK parameters were calculated using non compartmental methods with Phoenix WinNonlin software, version 6.1.0 (Pharsight, Cary, NC).

### Statistical analysis

Survival analysis, with footpad swelling as the measurement, was performed using the Kaplan-Meier method [27]. The log rank test was used to determine the level of statistical significance when comparing survival curves of the different treatment groups with the control group. *p* values were two-tailed, and a value of *p*<0.05 was considered statistically significant. CFU counts were log-transformed before analysis. Culture-negative footpads were assigned a log value of 0. Group means for experimental treatment groups were compared with that of the standard treatment control by one-way analysis of variance with Dunnett's post-test. Paired t-tests were also used to compare groups of equal size. All analyses were performed with GraphPad Prism version 4.01 (GraphPad, San Diego, CA).

## Results

### Checkerboard assay

The results of the checkerboard study are shown in table 1. They indicated that the interaction (ΣFIC = 0.75) between the two drugs was neither synergistic nor antagonistic and therefore was termed indifferent using the current guidelines [20], [21], [22].

**Table 1:** Checkerboard analysis for in-vitro interaction between clarithromycin (CLR) and rifampin (RIF).

	Alone	In-combination	FIC	ΣFIC
MIC of CLR	0.5	0.125	0.25	0.75
MIC of RIF	0.25	0.125	0.5	

FIC = MIC of the drug in combination÷MIC of the drug alone.

ΣFIC = FIC of CLR+FIC of RIF.

### CFU counts

The initial footpad suspension used for the inoculum contained 5.76 log_10_ CFU per ml or 4.24 log_10_ in the 0.03 ml that was inoculated per footpad. The next day (D1) 10 mice were sacrificed and the mean CFU count per footpad was 3.29±0.41 log_10_. On initiation of treatment, 11 days after infection (D11), the mean CFU count in the 10 mice that were sacrificed was 3.35±0.16 log_10_ CFU, indicating that there was no substantial multiplication in the footpads during the first 11 days.

**Figure 1:**
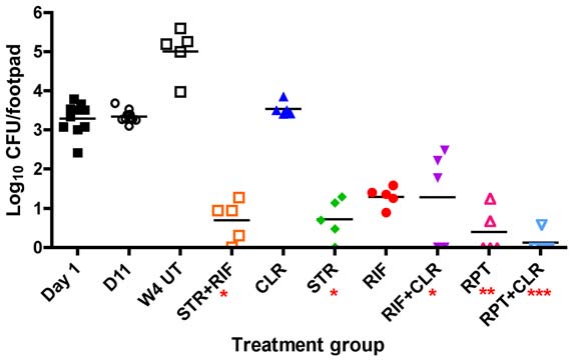
Reduction in footpad CFU counts with various treatment regimens. Mean CFU count from mouse footpads on the day after infection (Day1), the day of treatment initiation (D11), and after four weeks of: UT = no treatment, CLR = clarithromycin, RIF = rifampin, STR = streptomycin, RPT = rifapentine, The number of asterisks below the x-axis indicates the number of mice with negative footpad cultures.

On treatment completion (Figure 1), 4 weeks later, the mean log_10_ CFU count was 5.01±0.62 in untreated control mice (W4 UT), demonstrating that *M.ulcerans* had multiplied well, increasing by about 2 log_10_ in the footpads during the 4 weeks following treatment initiation, and suggesting a division time close to 4 days. In the positive control mice treated with STR+RIF, the mean log_10_ CFU count was 0.76±0.52, with one footpad out of the 5 harvested footpads culture-negative, underscoring the potent bactericidal activity of the STR+RIF combination against actively multiplying *M. ulcerans*. Among the test mice, the mean log_10_ CFU count was 3.54±0.18 in mice treated with CLR alone, a value similar to the 3.35±0.16 log_10_ value on treatment initiation, confirming the bacteriostatic activity of CLR against actively multiplying *M. ulcerans*. For other antibiotics alone or in combination, the mean (including footpads with negative culture) log_10_ CFU counts were significantly reduced (*p*<0.01) compared to the baseline value (Figure 1) but were not significantly different from each other except that mice treated with STR+RIF had a lower mean CFU count compared to RIF alone by paired t-test analysis (*p* = 0.0335, though not significant after adjustment for multiple comparisons): 0.82±0.58 for STR alone (no CFU was isolated from 1 of the 5 mice); 1.33±0.24 for RIF alone and 1.37±1.15 for RIF+CLR (no CFU was isolated from 1 of the 5 mice); 0.48±0.56 for RPT alone (no CFU was isolated from 2 of the 5 mice) and 0.20±0.31 for RPT+CLR (no CFU was isolated from 3 of the 5 mice).

### Time to footpad swelling

**Figure 2:**
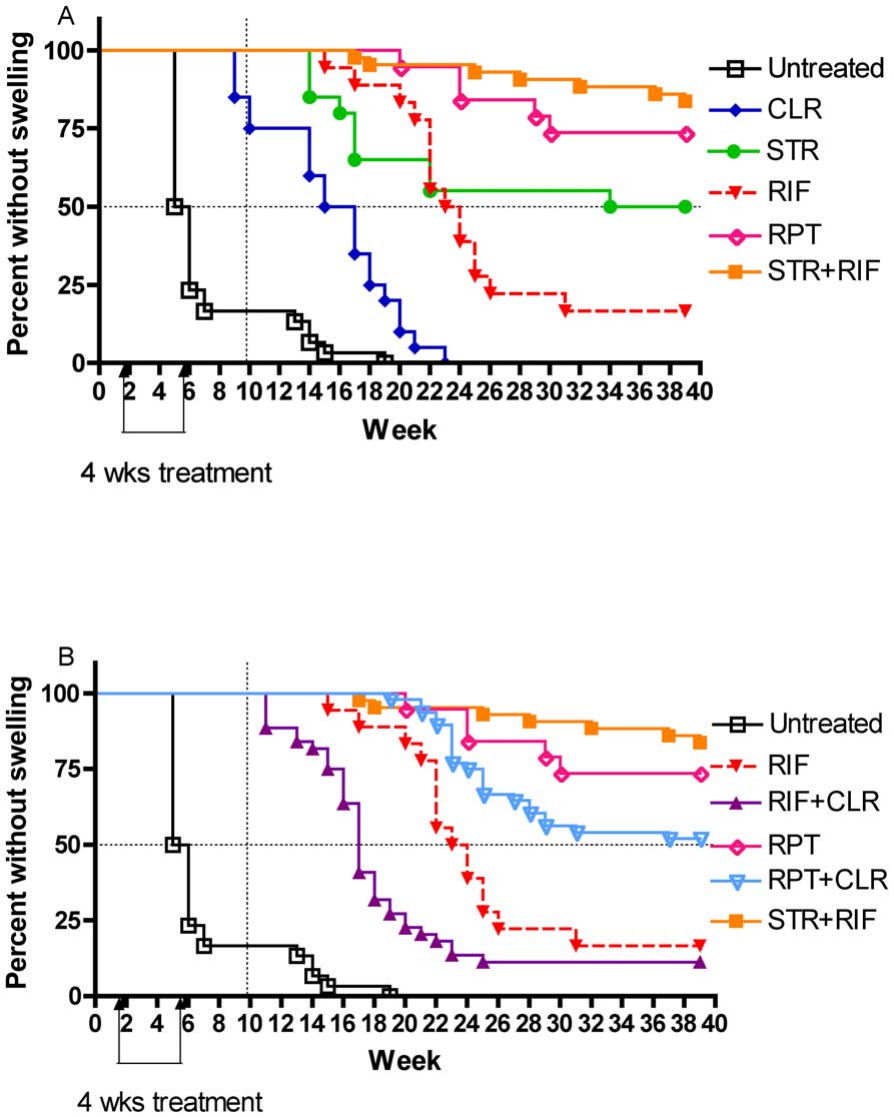
Delay in footpad swelling in mice receiving different drug regimens. Kaplan-Meier curve depicting the percentage of mice free of footpad swelling during 39 weeks of follow-up after infection. The dotted vertical line shows the expected delay in time to swelling for a bacteriostatic drug. The dotted horizontal line indicates footpad swelling in 50% of mice. A) Single drug regimens: Effect of single drugs compared to the standard regimen STR+RIF. Rifapentine alone prevented relapse as well as the standard STR+RIF regimen and better than any other single drug. B) Combination drug regimens with a rifamycin±CLR: Effect of adding CLR to either RIF or RPT. CLR = clarithromycin; RIF = rifampin; RPT = rifapentine, STR = streptomycin.

After completing 4 weeks of treatment, mice were monitored on a weekly basis for footpad swelling. Time to median swelling in untreated mice was 5 weeks after infection (Figure 2). In accordance with the CFU counts on treatment completion, mice treated with CLR were the first to reach footpad swelling. But the median time to swelling was 16 weeks after infection, well beyond the 9 weeks that would have been expected after 4 weeks of treatment with a purely bacteriostatic drug added to the 5 weeks time to swelling in untreated control mice. CLR treatment is thus accompanied by a prolonged delay in footpad swelling possibly due to a significant post-antibiotic effect. Mice treated by CLR were followed by mice treated by RIF alone and STR alone, with median time to footpad swelling of 23.5 and 34 weeks, respectively. Only 26.3% of mice treated with RPT alone and 11.4% of the positive controls treated with STR+RIF developed footpad swelling at the end of the 8-month follow-up period after treatment completion, emphasizing the potent sterilizing effect of both regimens. The difference between RPT alone and STR+RIF was not statistically significant (*p* = 0.33).

Surprisingly, as illustrated in Figure 2B, the time to footpad swelling was much shorter in mice treated with RIF+CLR (*p*<0.008) or RPT+CLR (*p* = 0.116) than in mice treated with RIF alone or RPT alone, respectively, suggesting antimicrobial or pharmacological antagonism between rifamycins and CLR in the mouse. Despite this antagonism, however, the RPT+CLR regimen caused a significantly greater (*p* = 0.0007) delay in footpad swelling than did RIF+CLR. Because the checkerboard study did not reveal antagonism between the two antimicrobials, the antagonism was likely to be due to pharmacokinetic drug-drug interaction as demonstrated below.

### Impact of CLR administration on rifamycin pharmacokinetics

**Figure 3:**
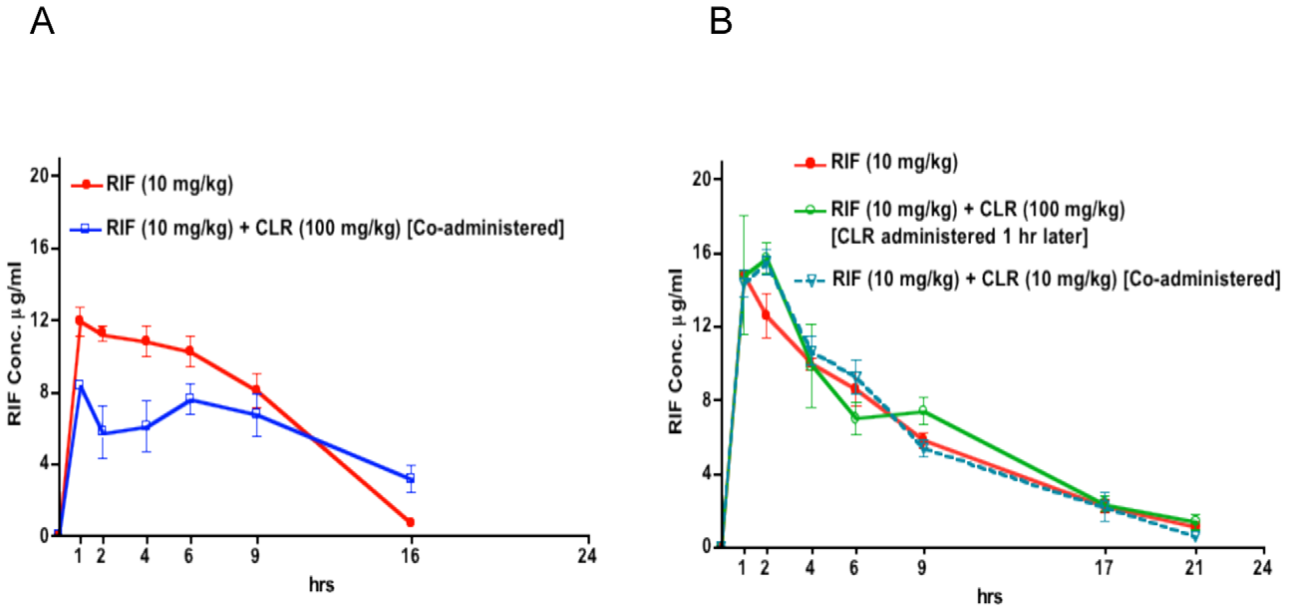
Effects of CLR administration on RIF concentrations. The reduction of serum RIF concentration by co-administered CLR is overcome by delayed CLR administration. RIF serum concentrations in mice after, A) RIF (10 mg/kg) given alone and co-administered with clarithromycin (CLR) (100 mg/kg), and; B) RIF given alone, with CLR (100 mg/kg) administered 1 hr later, or concurrently with CLR (10 mg/kg).

In the first PK study, as in the second and third PK studies, the experiments were performed as single and first-dose assessments, RIF (10 mg/kg) was either administered alone or co-administered with CLR (100 mg/kg). The mean AUC_0–16h_ and Cmax of RIF were 118.63±18 µg*hr/ml and 11.97±1.3 µg/ml, respectively, when RIF was administered alone, and 92.5±27 µg*hr/ml and 8.48±0.54 µg/ml, respectively, when RIF was co-administered with CLR (Figure 3A), suggesting that CLR co-administration led to diminished RIF concentrations. In a second PK study, RIF (10 mg/kg) was administered alone, with CLR (100 mg/kg) given 1 hr later, or co-administered together with CLR at a lower dose of 10 mg/kg. The mean AUC_0–21h_ and Cmax of RIF were 123±21 µg*hr/ml and 15.7±4.2 µg/ml, respectively, when RIF was administered alone, 133±32 µg*hr/ml and 16.7±2.5 µg/ml, respectively, when CLR (100 mg/kg) was administered 1 hr after RIF, and 125±22 µg*hr/ml and 15.5±1.2 µg/ml, respectively when RIF and CLR were co-administered at an equal dose of 10 mg/kg (Figure 3B).

**Figure 4:**
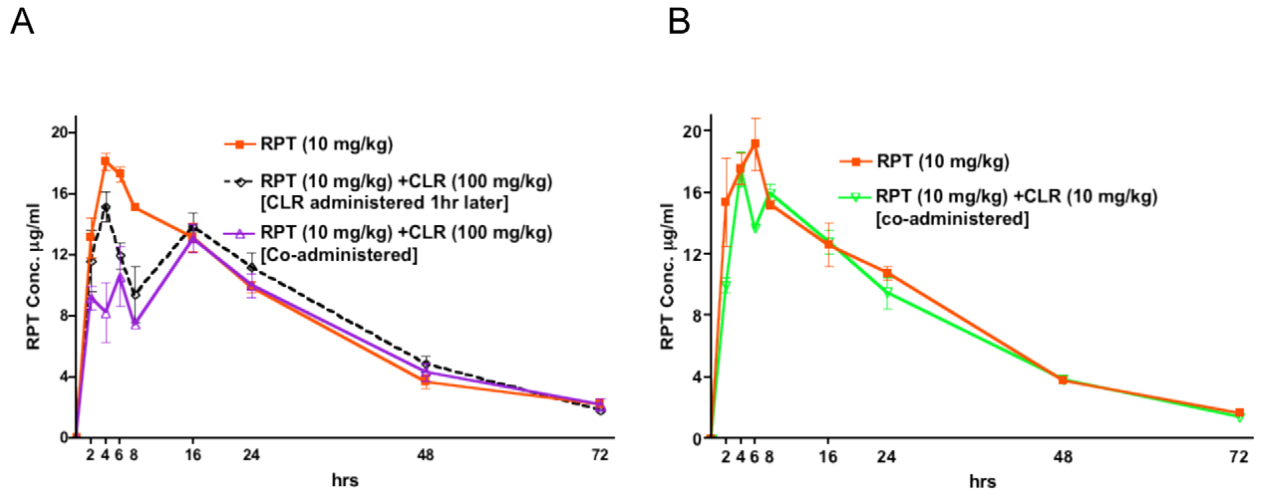
Effect of CLR administration on RPT concentrations. The reduction of serum RPT concentrations by co-administered CLA is overcome by delayed CLR administration. RPT serum concentrations in mice after, A) RPT (10mg/kg) given alone; RPT (10 mg/kg) followed by clarithromycin (CLR) (100 mg/kg) administered 1 hr later; or RPT (10 mg/kg) and CLR (100 mg/kg) concurrently; and B) RPT (10 mg/kg) given alone versus co-administered with lower-dose CLR (10 mg/kg).

Similar observations were made in mice given RPT and CLR. The AUC_0–24h_ and Cmax of RPT were moderately decreased from 317.24±25 µg*hr/ml and 18.11±1.0 µg/ml, respectively, when RPT was given alone to 241.09±0.37 µg*hr/ml and 13.07±1.6µg/ml, respectively, when RPT was co-administered with CLR (100 mg/kg). Delaying administration of CLR (100mg/kg) by 1 hr resulted in a RPT AUC_0–24h_of 279.70±0.47 µg*hr/ml and Cmax of 15.14±1.7 µg/ml (figure 4A). Reducing the dose of CLR to 10 mg/kg when co-administered with 10 mg/kg RPT resulted in RPT AUC_0–24h_and Cmax values of 302.51±28 µg*hr/ml and 17.52±1.1 µg/ml, respectively, similar to those of 324.23±44 µg*hr/ml and 19.24±1.6 µg/ml, respectively, obtained with RPT alone (Figure 4B).

## Discussion

The main result of the present work is that RPT alone administered 5 days a week at a dose of 10mg/kg is at least as active in terms of bactericidal effect and as active in terms of relapse prevention as the standard combination of STR+RIF against experimental *M.ulcerans* disease in the mouse. Such a result is extremely promising for the future of *M.ulcerans* disease treatment because it suggests that an entirely oral treatment may be as active as the present regimen containing parenteral STR. However, RPT cannot be administered alone because of the risk of drug resistance resulting from monotherapy, and CLR is the oral companion drug of choice to combine with a rifamycin [28]. As CLR alone exhibited clear-cut bacteriostatic activity, an additive effect of the combination RPT+CLR and even RIF+CLR was expected. Unfortunately the co-administration of a rifamycin and CLR, both drugs given orally at doses equivalent to human doses on the basis of serum AUC, was less effective than each rifamycin alone in mice infected with *M. ulcerans*. As no antagonistic effect between RIF and CLR was exhibited *in vitro* in the checkerboard assay, the lesser *in vivo* effectiveness of the combination could not be related to a negative antimicrobial drug-drug interaction. Rather, it appears that the negative drug-drug interaction was pharmacokinetic in nature. Indeed, co-administration of a 10-mg/kg dose of RIF with a 100 mg/kg dose of CLR resulted in a 22% reduction of RIF AUC and a 29% reduction of RIF Cmax compared to administration of RIF alone. Similarly, RPT 10 mg/kg given together with 100 mg/kg CLR resulted in a 24% and 28% reduction of the RPT AUC and Cmax, respectively, compared to RPT administered alone. When administration of 100mg/kg of CLR was delayed by one hour from RIF administration, the pharmacokinetic interaction became insignificant. Similarly, there were also no negative pharmacokinetic drug-drug interactions when mice were co-administered 10 mg/kg of RIF and 10 mg/kg of CLR. These results indicate that the co-administration of CLR and RIF negatively interacts with the blood levels of rifamycins in mice probably by interfering with their absorption or by another mechanism and that this drug interaction is dose-dependent. However, a recent study in humans showed that concomitant CLR did not impact the absorption rate constant, the Cmax, or the Tmax of RIF at steady state, indicating that, at clinically relevant doses (7.5 mg/kg of CLR and 10 mg/kg of RIF), CLR does not negatively affect the levels of rifamycins in humans [29].

Our findings illustrate the difficulties in designing experiments in the murine model that aim to instruct treatment of a human infectious disease and in interpreting their results.

In order to adequately assess in mice the antimicrobial potential of a given drug, that drug should be given at doses deemed equivalent to human doses. As, drugs are usually metabolized much more rapidly in mice than in humans, the drug doses in mice have to be increased to obtain similar drug exposure in mice as in humans [25], [30]. That is the case for CLR [30], [31]. But the dose of 100 mg/kg that is adequate in mice to assess the antimicrobial activity of CLR when the drug is used alone presents a problem when it is co-administered with 10 mg/kg of RIF, most likely by reducing the absorption of RIF. Therefore the fact that combinations of CLR and a rifamycin were less active than the corresponding rifamycin alone should be considered an experimental artifact, and both drugs should be administered separately, with an interval of no less than one hour between them. Interestingly, the same phenomenon is observed in the experimental chemotherapy of tuberculosis for which RIF should be administered at least one hour before isoniazid and pyrazinamide to prevent a negative pharmacokinetic interaction in mice [32], [33].

Although the negative pharmacokinetic interactions prevented a reliable assessment of the antimicrobial activity of the RIF+CLR and RPT+CLR combinations against experimental *M.ulcerans* disease in mice, they did not prevent assessment of each drug alone in reference to the positive controls receiving STR+RIF. Besides the promising potency of RPT, our study also emphasizes the peculiar prolonged delay in footpad swelling resulting from treatment with CLR in mice infected with *M. ulcerans*. Whatever its antimicrobial or immunomodulatory [34] nature, this delay in footpad swelling is favorable and supports the use of CLR in the treatment of Buruli ulcer.

Finally, it is important to note that, in our experimental model, drug activity was assessed during a 4-week period during which untreated animals had a 2 log_10_ increase in CFU counts in their footpads. As expected, CLR exhibited bacteriostatic activity whereas other drugs exhibited bactericidal activity, especially RPT. But, very interestingly, even the most active drugs and drug regimens did not reduce the CFU counts by more than 3 log_10_ in 4 weeks. This was much less than the 5–6 log_10_ reduction in the CFU counts observed by Ji et al. [12], [13], [14] when treatment was initiated at the plateau phase of growth, i.e., when the organisms were no longer actively multiplying likely because of immune containment. In our experimental model because antibiotic treatment was initiated during the incubation phase of the disease, the reduction in the CFU counts and the time to foot pad swelling are measuring only the antimicrobial activity. When treatment is initiated at the plateau phase of growth, its effect is likely a mixture of antimicrobial activity, immune containment, and shutting down the enzymes involved in mycolactone production. It does not facilitate the assessment of the respective antimicrobial value of each drug regimen, even though it might better recapitulate the response of patients to antibiotic therapy. In the chemotherapy of BU, as in the chemotherapy of tuberculosis, the drug activity against actively multiplying organisms, usually termed bactericidal activity, is very different from the drug activity against organisms that are no longer actively multiplying, i.e., sterilizing activity. Consequently, the occurrence, magnitude, and duration of the antimicrobial effect depend on the experimental model used. The information provided by each model is therefore different yet complementary, and not at all contradictory.
